# Integrated network pharmacology and comprehensive bioinformatics analysis to identify the mechanisms and molecular targets of ketamine in ischemic stroke–depression comorbidity

**DOI:** 10.1371/journal.pone.0343918

**Published:** 2026-03-18

**Authors:** Yunfei Shu, Yinhao Guo, Suihan Xu, Hongxia He, Biao Zeng, Zhenyu Yang, Wei Gao, Jun Li

**Affiliations:** 1 Mianyang Key Laboratory of Anesthesia and Neuroregulation, Department of Anesthesiology, Mianyang Central Hospital, Mianyang, China; 2 Department of Anesthesiology, The First Affiliated Hospital of Xi’an Jiaotong University, Xi’an, People’s Republic of China; Nathan S Kline Institute, UNITED STATES OF AMERICA

## Abstract

Comorbid depression following ischemic stroke is a debilitating condition with complex pathophysiology. Although ketamine demonstrates rapid antidepressant effects, its molecular mechanisms in the context of this comorbidity are poorly understood. We employed a network pharmacology approach to hypothesize potential molecular targets and pathways of ketamine relevant to both ischemic stroke and depression. Differentially expressed genes for ischemic stroke and major depressive disorder were identified from public Gene Expression Omnibus datasets. A broad set of potential ketamine-associated genes was compiled from the SwissTargetPrediction, Comparative Toxicogenomics Database, and GeneCards databases. The intersection of these gene sets was analyzed via Gene Ontology and Kyoto Encyclopedia of Genes and Genomes enrichment analyses. A protein–protein interaction network was constructed, and hub genes were identified. SHAP-based interpretability analysis was performed to rank the relative importance of these hub genes. Further analyses, including gene set enrichment analysis, immune infiltration estimation, and signaling network reconstruction, were conducted to characterize the functional context of key candidates. Molecular docking was performed to probe potential interactions between ketamine and candidate proteins. Our integrative analysis identified 42 intersecting genes, with enrichment in pathways such as lipid and atherosclerosis, interleukin-17 signaling, and cellular response to stimuli. Two genes, IL1RN and DDIT3, emerged as central candidates and were linked to immune regulation, neurotrophin signaling, and ubiquitin-mediated processes in our subsequent analyses. Docking simulations suggested potential binding of ketamine to these proteins. These in silico findings propose that the putative effects of ketamine on comorbid ischemic stroke and depression may involve modulation of multiple pathways, with IL1RN and DDIT3 as potential key contributors. This work provides a hypothesis-generating framework for future experimental and clinical validation.

## Introduction

Stroke is the second leading cause of death worldwide and a major contributor to disability, imposing a substantial burden on families and society [1]. Post-stroke complications are common and include motor and physical impairments as well as neuropsychiatric conditions such as anhedonia, depression, mania, and other psychological disorders [2,3]. Post-stroke depression (PSD) is a frequent and debilitating sequela of acute stroke, characterized by anxiety, anhedonia, social dysfunction, and hopelessness [4]. Approximately one-third of stroke survivors are diagnosed with depression within 5 years [5,6], and symptoms often persist for at least 1 year [7]. Depression following stroke severely hampers post-stroke recovery [8], particularly of cognitive and motor functions, and negatively impacts the quality of life [9,10]. The co-occurrence of depression and stroke is thus a major clinical challenge, and early identification and intervention are essential for patient rehabilitation [11].

The pathogenesis of comorbid depression and stroke remains incompletely understood but is believed to involve a complex interplay of psychosocial factors, genetic predisposition, biological mechanisms, and neural circuitry changes [8,12]. Studies have indicated associations with alterations in the expression of brain-derived neurotrophic factor (BDNF) and its receptor TrkB, which are crucial for neuronal development and synaptic plasticity regulation. Post-stroke dysfunctions in BDNF expression may disrupt neurotransmitter balance, impair neural signal transduction, and promote depressive symptoms [13]. Additionally, binding of the BDNF precursor (proBDNF) to the p75NTR receptor can activate the RhoA–JNK signaling pathway, inducing apoptosis-related protein expression, which has been implicated in neuronal loss and impaired plasticity and may contribute to post-stroke neuropsychiatric sequelae [10]. In a hemorrhagic mouse model, structural and functional abnormalities in the frontal lobe circuitry and JAK–STAT signaling pathway dysregulation were associated with depressive-like behaviors; thus, targeting this pathway may reduce severity and provide therapeutic effects [8]. Qinlin et al. [7] suggested that hippocampal damage after stroke may involve multiple signaling pathways and increased expression of autophagy-related genes, based on differential gene analysis in a rodent model. Primary cortical ischemia may induce secondary hippocampal damage, upregulating miR34b-3p expression in neurons, suppressing eIF4E, impairing neurotransmitter-related protein synthesis, damaging neuroplasticity, and inducing neuronal apoptosis, potentially leading to depressive outcomes [11]. Collectively, these findings suggest that the pathophysiology linking stroke and depression involves neurotransmitter imbalances, neural circuit alterations, synaptic plasticity impairments, and neuroinflammation.

Current antidepressant treatments for depression in stroke survivors have limitations, including delayed onset of action and potential adverse effects on the nervous and cardiovascular systems [14,15]. Selective serotonin reuptake inhibitors (SSRIs), a common first-line treatment, typically require weeks to take effect [16,17]. Furthermore, approximately 50% of patients experience partial relief and 30% achieve symptom remission with SSRIs [2]. However, SSRIs have been associated with increased bleeding risk and, in some studies, cardiovascular events. A clinical study reported that early administration of escitalopram for post-stroke depressive symptoms did not reduce the number of patients with moderate to severe symptoms but increased that of patients with no or mild symptoms [18]. Another study reported that administering fluoxetine (20 mg/day) for 6 months after acute stroke reduced the incidence of depression but failed to improve neurological outcomes and increased fracture frequency [3,19]. These limitations underscore the need for novel therapeutic approaches with different mechanisms of action.

Ketamine, an N-methyl-D-aspartate receptor (NMDAR) antagonist, has demonstrated rapid and sustained antidepressant effects in major depressive disorder [3,20,21]. It directly modulates glutamatergic and GABAergic neurotransmission [22]. Its rapid antidepressant action may involve NMDAR inhibition on GABAergic interneurons, leading to the transient disinhibition of glutamatergic pyramidal neurons and increased glutamate release in regions such as the prefrontal cortex (PFC) and hippocampus. This, in turn, activates AMPA receptors (AMPAR), triggers signaling cascades such as BDNF–TrkB–ERK and PI3–AKT–mTOR, and may enhance synaptogenesis and synaptic plasticity [23]. Concurrently, NMDAR blockade by ketamine can deactivate eEF2K, reducing eEF2 phosphorylation and increasing BDNF translation. BDNF signaling appears critical, as disruption of BDNF–TrkB signaling in the medial PFC abolishes ketamine’s antidepressant effects [24]. Furthermore, ketamine has been reported to interact with μ, κ, and δ opioid receptors, potentially contributing to its effects in treatment-resistant depression (TRD) [25]. Its sustained effects may involve the promotion of synaptic plasticity [23]. In a rat model combining middle cerebral artery occlusion and chronic unpredictable mild stress, ketamine induced rapid and persistent antidepressant-like effects, potentially by regulating the NMDAR–CaMKII signaling pathway to promote synaptic plasticity [3]. Despite this promising profile, the molecular targets and pathways through which ketamine might exert effects in the specific context of comorbid ischemic stroke and depression remain largely unexplored.

Network pharmacology, an emerging field, shifts the focus from single- to multi-target approaches by integrating systems biology and computational analysis. It encompasses drug and disease target identification, protein–protein interaction (PPI) network analysis, key gene screening, and functional enrichment studies, and has been increasingly applied to investigate the mechanisms of drug action [26]. Therefore, in this study, we aimed to employ a network pharmacology approach to hypothesize potential molecular targets and pathways of ketamine relevant to both ischemic stroke and depression. By integrating public high-throughput sequencing data with computational analyses, we sought to generate a testable framework for understanding ketamine’s putative role in this complex comorbidity, which may inform future experimental and clinical research.

## Materials and methods

### Data acquisition

The datasets used in this study were sourced from the National Center for Biotechnology Information Gene Expression Omnibus (GEO) database [27] (https://www.ncbi.nlm.nih.gov/) (Table 1).

**Table 1:** National center for biotechnology information gene expression omnibus datasets information.

		Accession ID	Disease	Control	Platform
**Ischemic stroke**	Training	GSE16561	39	24	GPL6883
	Validation	GSE58294	69	23	GPL570
**Depression**	Training	GSE23848	20	15	GPL6106
	Validation	GSE76826	20	12	GPL17077

### Data preprocessing and gene annotation

Preprocessed, normalized, and log2-transformed probe expression matrices were downloaded from the GEO database, along with platform annotation files. Probes without matching gene symbols were excluded. For multiple probes mapping to the same gene, the median value across probes was used as the final gene expression value.

### Differential analysis

Differential gene expression analysis was performed using the limma package [28] (version 3.10.3, http://www.bioconductor.org/packages/2.9/bioc/html/limma.html) on the ischemic stroke (GSE16561) and depression (GSE23848) training sets, comparing disease (disease group) vs. normal control (control group) samples. To capture a broader spectrum of expression changes for subsequent intersection analysis, genes with a nominal p-value <0.05 and |logFC| > 0.2 were initially designated as differentially expressed genes (DEGs) (thresholds based on PMID: 34786719; PMID: 39858215; PMID: 39222930; PMID: 38684463), yielding DEGs for ischemic stroke (DEGs1) and depression (DEGs2). The results were visualized using volcano plots and expression heatmaps with the R package ggplot2 (version 3.4.4; https://github.com/tidyverse/ggplot2).

### Prediction of putative ketamine targets

Prediction of putative ketamine targets was compiled from the Comparative Toxicogenomics Database (CTD) [29] and GeneCards [30] databases. The CTD (http://ctdbase.org/) is a comprehensive database focused on chemical-gene-disease interactions. GeneCards (https://www.genecards.org/), developed by the Weizmann Institute of Science, integrates multiple bioinformatics resources to provide detailed gene annotations. Additionally, the chemical structure of ketamine was retrieved from the PubChem database (https://pubchem.ncbi.nlm.nih.gov) and submitted to the Swiss Target Prediction database (http://swisstargetprediction.ch). Targets were screened using a probability threshold of ≥0.1 to select relevant genes. The target genes from these sources were combined, duplicates were removed, and the resulting set was designated as the putative ketamine targets.

### Identification of differentially expressed putative ketamine targets

To identify putative ketamine targets that were also differentially expressed in ischemic stroke and depression, Venn diagrams were employed to determine the intersections among DEGs1, DEGs2, and putative ketamine targets. The resulting genes were defined as the intersection genes and used for subsequent analyses.

### GO and KEGG analyses of the intersection genes

Based on the intersection genes, GO [31] and KEGG [32] enrichment analyses were conducted using the R package clusterProfiler [33] (https://github.com/YuLab-SMU/clusterProfiler). Results with an adjusted p-value <0.05 were considered significantly enriched and visualized using the R package ggplot2.

### PPI network construction

The STRING database (version 12.0; http://www.string-db.org/) [34] was used to predict potential interactions among the proteins encoded by the intersection genes. The input gene set consisted of the intersection genes, with the species set to *Homo sapiens* and the PPI score threshold set at 0.4, yielding a PPI network that was visualized using Cytoscape software (version 3.7.2, https://cytoscape.org/). Hub genes were identified using the cytoHubba plugin, employing the maximal clique centrality (MCC), Node Degree, edge percolated component (EPC), and maximum neighborhood component (MNC) algorithms. The top 15 genes from each algorithm were selected, and their intersection was considered as the hub genes.

### SHAP-based interpretability analysis and feature ranking

An Extreme Gradient Boosting (XGBoost) model was developed using the R package xgboost [35] (1.7.8.1, https://cran.r-project.org/web/packages/xgboost/index.html) based on feature genes within the training sets of the two diseases. Subsequently, the model was interpreted using the R package shapviz [36] (0.9.7, https://github.com/ModelOriented/shapviz), and SHAP values for each feature gene were calculated.

### Candidate biomarker identification

To determine the expression profiles of hub genes in disease vs. control samples, their expression consistency was analyzed across training and validation sets. ROC curves for hub genes were computed using the R package pROC (version 1.18.5, https://xrobin.github.io/pROC/) in training and validation sets. Genes exhibiting significant expression differences and an AUC > 0.7 were designated as candidate biomarkers. Correlations among candidate biomarkers were visualized using correlation heatmaps.

### GSEA

To explore potential molecular mechanisms associated with the candidate biomarkers, single-gene GSEA was performed using the R package clusterProfiler, based on the MSigDB database (www.msigdb.org) [37] using the reference gene set c2.cp.kegg_legacy.v2024.1.Hs.symbols.gmt. This analysis identified signaling pathways potentially associated with each biomarker in the training sets.

### Association between candidate biomarkers and immune infiltration

To investigate immune infiltration in ischemic stroke and depression, the CIBERSORT algorithm within the R package IOBR (version 0.99.0, https://github.com/IOBR/IOBR) was applied to assess the abundance of 22 immune cell types in disease and control samples. Differences in immune cell infiltration were analyzed, and immune cells exhibiting significant intergroup differences were identified. Pearson correlation tests were conducted to explore correlations between differentially infiltrated immune cells and biomarkers in disease samples.

### SIGNOR analysis of biomarkers

To analyze potential upstream–downstream relationships of the candidate biomarkers and identify regulatory entities, the SIGNOR 3.0 (https://signor.uniroma2.it) [38] was utilized. SIGNOR provides an accessible static map of causal interactions, which can be customized, refined, and expanded to construct dynamic and predictive models. Each signaling relationship is annotated with its effect (upregulation or downregulation) and mechanism (e.g., binding, phosphorylation, transcriptional activation) influencing the target entity.

### Molecular docking

The three-dimensional (3D) molecular structure of ketamine was retrieved from the PubChem database (https://pubchem.ncbi.nlm.nih.gov/) [39], and the corresponding 3D protein structures of candidate biomarkers were obtained from the AlphaFold database (https://alphafold.ebi.ac.uk/). Molecular docking between ketamine and biomarkers was performed using CBdock (https://cadd.labshare.cn/cb-dock2/php/index.php) [40], with results visualized using PyMOL (https://pymol.org/) [41].

## Results

### Differential analysis

**Fig 1:**
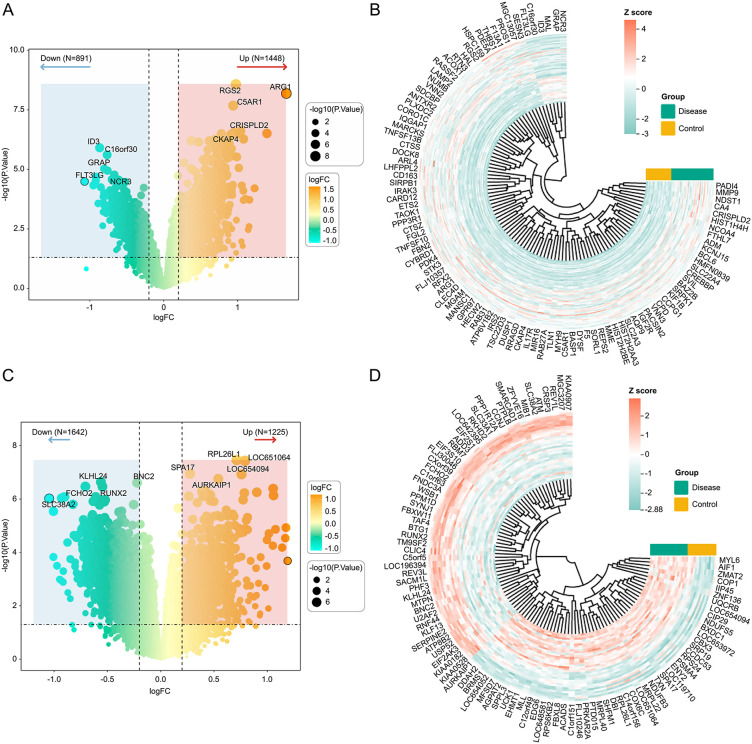
Identification of differentially expressed genes in ischemic stroke and depression compared with normal controls. (a) x- and y-axes denote logFC gene expression fold change and -log₁₀(p-value) statistical significance, respectively. Negative and positive logFC indicate downregulation and upregulation, respectively. **(b)** Heatmap of the top 100 differentially expressed genes. (c) x- and y-axes denote logFC gene expression fold change and -log₁₀(p-value) statistical significance, respectively. Negative and positive logFC indicate downregulation and upregulation, respectively. **(d)** Heatmap of the top 100 differentially expressed genes.

Differential analysis was conducted using the R package limma on the ischemic stroke (GSE16561) and depression (GSE23848) training sets, with thresholds of p-value <0.05 and |log₂FC| > 0.2. In the ischemic stroke training set, 1,448 and 891 upregulated and downregulated genes were identified, respectively (Fig 1a, 1b); in the depression dataset, 1,225 and 1,642 upregulated and downregulated genes were identified, respectively (Fig 1c, 1d).

### Identification of differentially expressed putative ketamine targets

**Fig 2:**
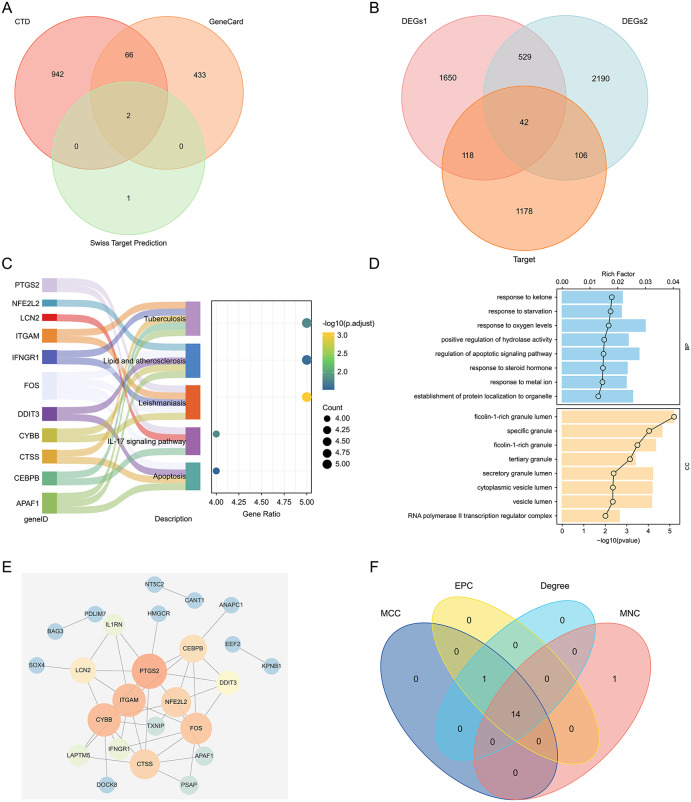
Identifying key hub genes through target mapping, functional enrichment, and network analysis. **(a)** Potential ketamine targets obtained from the CTD, GeneCards, and Swiss Target Prediction database. **(b)** Venn diagram showing the intersections among DEGs1, DEGs2, and putative ketamine targets. **(c)** Kyoto Encyclopedia of Genes and Genomes (KEGG) enrichment results for intersection genes. Sankey diagram shows gene–pathway relationships. Dot plot x- and y-axes represent the number of enriched genes and enriched KEGG pathways, respectively. Dot size and color indicates gene count and p-value magnitude, respectively. **(d)** GO enrichment results for intersection genes. The top 8 results per category are shown. **(e)** Protein interaction network of intersection genes. Node size and color indicate Degree: larger Degree values signify greater importance within the network. **(f)** Venn diagram of feature gene intersections identified by the MCC, Degree, EPC, and MNC algorithms.

Putative ketamine targets were obtained from the CTD, GeneCards, and Swiss Target Prediction database, which yielded 1,010, 501, and 3 target genes, respectively; these results were pooled as a set of 1,444 target genes (Fig 2a).

To identify differentially expressed target genes related to ketamine in ischemic stroke and depression, intersection genes were identified among DEGs for ischemic stroke (DEGs1) and depression (DEGs2) training sets, as well as the putative ketamine targets, yielding 42 intersecting genes for subsequent analysis (Fig 2b).

### Enrichment analysis

To investigate the potential biological mechanisms and signaling pathways associated with the intersecting genes, Gene Ontology (GO) and Kyoto Encyclopedia of Genes and Genomes (KEGG) enrichment analyses were performed. KEGG analysis suggested that the intersecting genes were primarily enriched in pathways related to “Tuberculosis,” “Lipid and Atherosclerosis,” “Leishmaniasis,” “IL-17 Signaling Pathway,” and “Apoptosis” (Fig 2c). GO analysis indicated enrichment in biological processes including “Response to Ketone Bodies,” “Response to Starvation,” and “Response to Oxygen Levels.” In cellular components such as “Ficolin-1-Rich Granule Lumen,” “Specific Granule,” and “Ficolin-1-Rich Granule,” no significant enrichment was observed in molecular functions (Fig 2d).

### PPI network construction

A PPI network was constructed based on the 42 intersecting genes (Fig 2e). Feature genes were screened using the MCC, MNC, EPC, and Degree algorithms within the cytoHubba plugin, selecting the top 15 genes from each algorithm. The intersection of these four sets yielded 14 hub genes (Fig 2f).

### SHAP-based interpretability analysis and feature ranking

**Fig 3:**
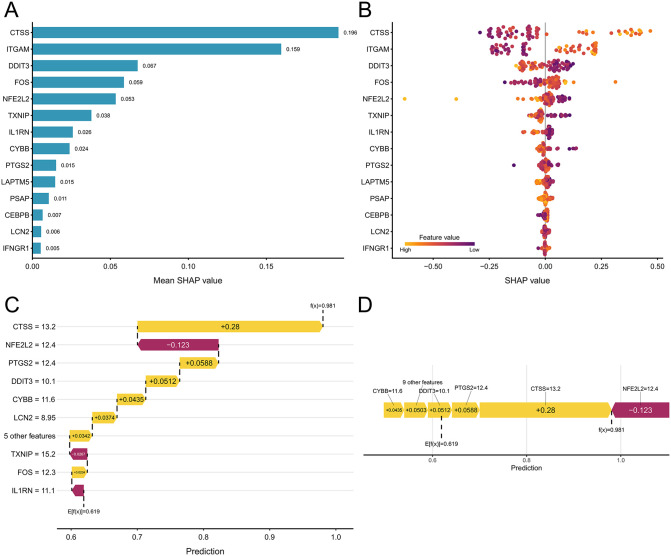
SHAP-based interpretability analysis and feature ranking for the ischemic stroke model (GSE16561). **(a)** Bar plot of the SHAP-based feature importance ranking in the ischemic stroke model, with genes ordered by descending mean absolute SHAP value. **(b)** Beeswarm plot illustrating the distribution and impact direction of SHAP values for the top 14 features in the ischemic stroke model, ordered vertically by descending mean absolute SHAP value. **(c)** Waterfall plot decomposing an individual ischemic stroke prediction into additive SHAP contributions from the top features, starting from the baseline E[f(x)] = 0.619. **(d)** Force plot illustrating how feature contributions collectively shift an individual ischemic stroke prediction from the baseline E[f(x)] = 0.619 to the final output f(x) = 0.981.

In the ischemic stroke model (GSE16561), CTSS was identified as the most important feature. This was followed by ITGAM, DDIT3, FOS, NFE2L2, TXNIP, IL1RN, CYBB, and PTGS2, all of which contributed substantially to the model output. In contrast, the contributions of LCN2 (0.006) and IFNGR1 (0.005) were relatively minor. Overall, the model’s predictions were largely influenced by a small set of immune- and inflammation-related genes (Fig 3a).

The beeswarm plot (Fig 3b) revealed considerable heterogeneity in the contributions of individual features to the model output across samples. CTSS and ITGAM yielded large positive SHAP values in a subset of samples, suggesting a strong contribution to high-risk predictions in those samples. Several genes (e.g., NFE2L2, TXNIP, IL1RN) exhibited directional differences in their SHAP values across their value ranges, indicating potential nonlinear relationships with the model output or influences from feature interactions.

The waterfall plot provided an explanation for a representative individual (Fig 3c, 3d). The main features contributing positively to elevating the predicted risk were: CTSS: + 0.280, PTGS2: + 0.0588, DDIT3: + 0.0512, CYBB: + 0.0435, and LCN2: + 0.0374. Conversely, several features exerted suppressive effects on the risk prediction: NFE2L2: −0.123, TXNIP: −0.0267, and IL1RN: −0.0181.

**Fig 4:**
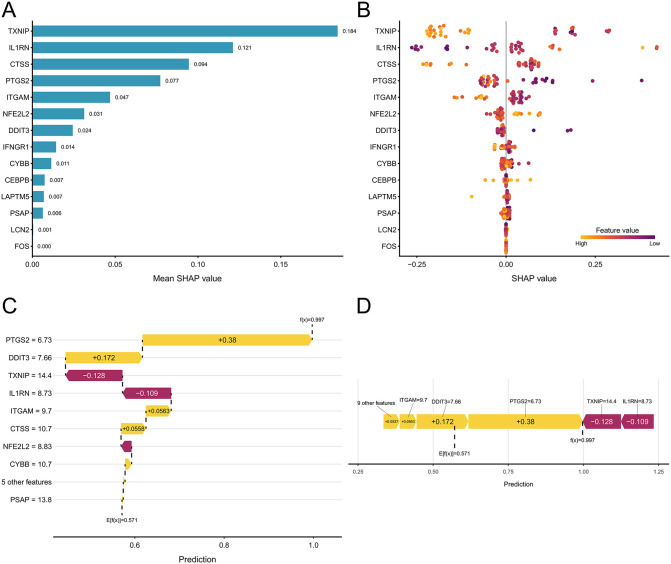
SHAP-based interpretability analysis and feature ranking for the depression model (GSE23848). **(a)** Bar plot of the SHAP-based feature importance ranking in the depression model, with genes ordered by descending mean absolute SHAP value. **(b)** Beeswarm plot showing the distribution and direction of SHAP values for the top 14 features in the depression model, ordered vertically by descending mean absolute SHAP value. **(c)** Waterfall plot decomposing an individual depression model prediction into additive SHAP contributions, starting from the baseline E[f(x)] = 0.571. **(d)** Force plot illustrating how feature contributions collectively shift an individual depression model prediction from the baseline E[f(x)] = 0.571 to the final output f(x) = 0.997.

In the depression model (GSE23848), TXNIP was the primary feature, followed by IL1RN, CTSS, PTGS2, ITGAM, NFE2L2, and DDIT3. In contrast, the global contributions of LCN2 (0.001) and FOS (0.000) were minimal, suggesting limited discriminatory power in this model or that their information was subsumed by other features (Fig 4a).

The beeswarm plot (Fig 4b) shows that features such as TXNIP and IL1RN in the depression model exhibit more pronounced SHAP value distributions, indicating a more consistent directional influence (pushing or suppressing predictions) across samples. Meanwhile, features like CTSS, PTGS2, and ITGAM still demonstrate certain nonlinear patterns and sample heterogeneity, aligning with the model’s capacity to capture complex relationships.

The waterfall plot (Fig 4c, 4d) for a representative sample shows a baseline prediction E[f(x)] of 0.571 and a final output f(x) of 0.997. The main positive driving forces were PTGS2: + 0.38, DDIT3: + 0.172, ITGAM: + 0.0563, and CTSS: + 0.0558. The primary negative contributions came from TXNIP: −0.128 and IL1RN: −0.109. Contributions from other features (e.g., NFE2L2, CYBB, PSAP) were minor. This individual explanation indicates that although TXNIP and IL1RN exerted substantial suppressive effects on the risk, the strong positive pushes from PTGS2 and DDIT3 were dominant, resulting in a final prediction close to 1.

### Candidate biomarker identification

**Fig 5:**
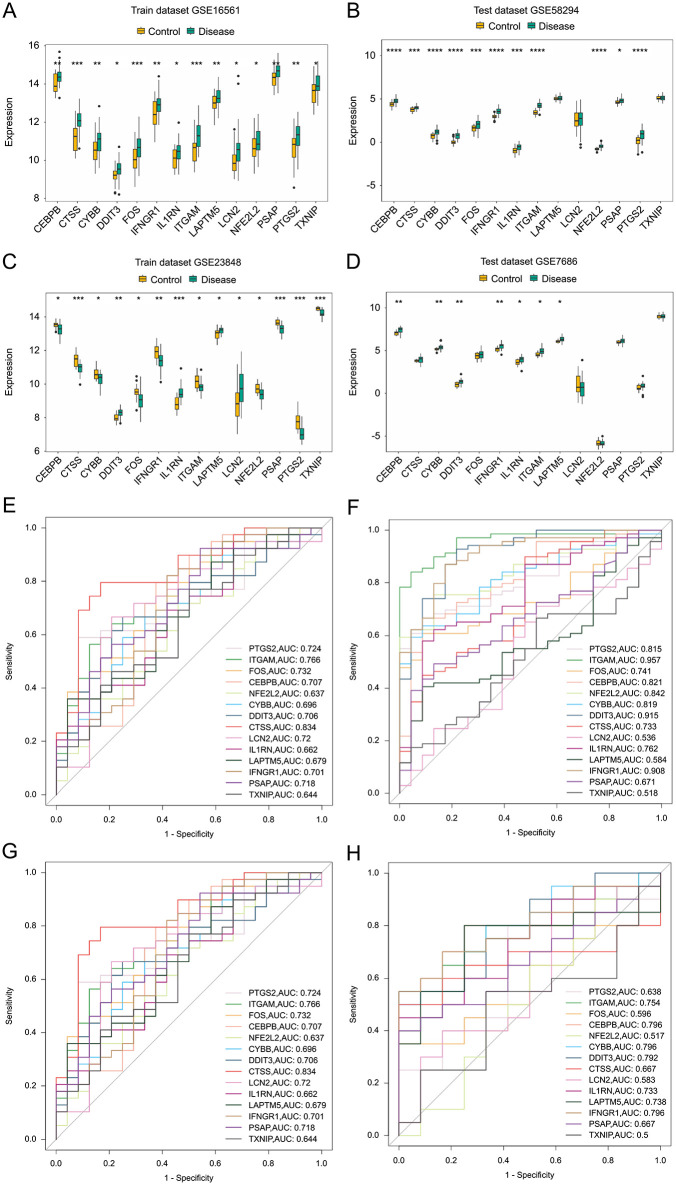
Expression of hub genes in ischemic stroke training(a) GSE16561 and validation (b) GSE58294 sets. X- and y-axes represent genes and expression levels, respectively. p-values were estimated by the Wilcoxon rank-sum test: *p < 0.05, ***p < 0.001, ****p < 0.0001). Expression of hub genes in depression training **(c)** (GSE23848) and validation **(d)** (GSE76826) sets. X- and y-axes represent genes and expression levels, respectively. p-values were estimated by the Wilcoxon rank-sum test: *p < 0.05, **p < 0.01, ***p < 0.001, ****p < 0.0001). Receiver operating characteristic (ROC) curves of hub genes in ischemic stroke training **(e)** GSE16561 and validation **(f)** GSE58294 sets. X- and y-axes represent false and true positive rates, respectively. ROC curves of feature genes in depression training **(g)** GSE23848 and validation **(h)** GSE76826 sets. X- and y-axes represent false and true positive rates, respectively.

**Fig 6:**
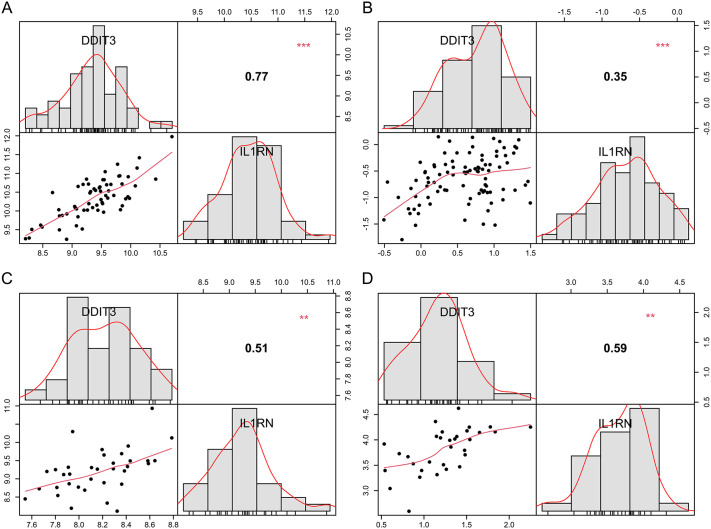
Correlation among biomarkers in (a) ischemic stroke training and (b) validation sets. (c) and (d) show depression training and validation sets, respectively. Correlation coefficients and p-values were derived from the Pearson correlation test.

The expression profiles of the 14 hub genes were analyzed in the disease and control groups across training and validation sets for both diseases. *DDIT3* and *IL1RN* genes exhibited consistent and significant expression trends in both sets (Fig 5a–5d). Their ROC curve AUC values exceeded 0.7 in three of the four datasets, except in the ischemic stroke training set where their AUC was < 0.7 (Fig 5e–5h). Hence, the *DDIT3* and *IL1RN* genes demonstrated high correlation (Fig 6a–6d), thereby defining them as candidate biomarkers.

### Gene set enrichment analysis (GSEA)

**Fig 7:**
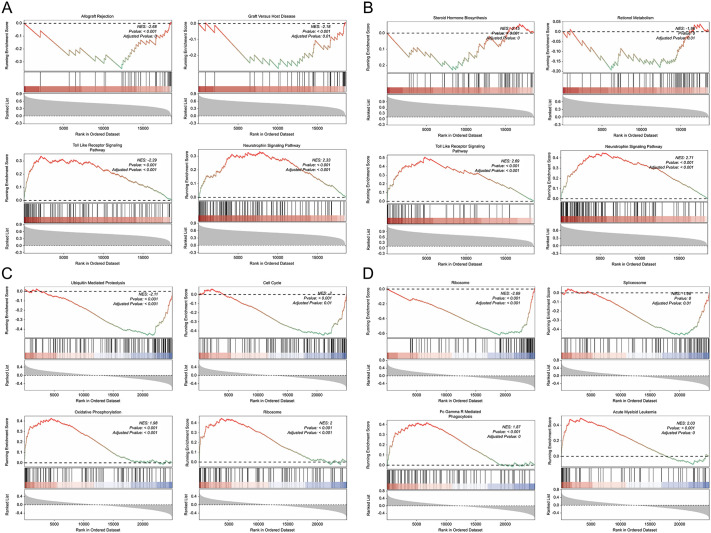
Gene set enrichment analysis (GSEA) of DDIT3 and IL1RN reveals significant pathway associations in ischemic stroke and depression. **(a)** GSEA results for DDIT3 in ischemic stroke. The two highest and lowest enriched pathways are shown. Line plot shows enrichment score (ES) for ranked gene sets, barcode plot marks positions of pathway gene set members in the ranked gene list, and heatmap shows the distribution of rank values for all genes post-ranking. **(b)** GSEA enrichment results for IL1RN in ischemic stroke. The two highest and lowest enriched pathways are shown. Line plot shows ES, barcode plot marks gene positions, and heatmap shows rank value distribution. **(c)** GSEA enrichment results for DDIT3 in depression. The two highest and lowest enriched pathways are shown. Line plot shows ES, barcode plot marks gene positions, and heatmap shows rank value distribution. **(d)** GSEA enrichment results for IL1RN in depression. The two highest and lowest enriched pathways are shown. Line plot shows ES, barcode plot marks gene positions, and heatmap shows rank value distribution.

GSEA was performed to further elucidate the molecular mechanisms of the biomarkers. In ischemic stroke, DDIT3 was primarily associated with signaling pathways such as “Allograft Rejection,” “Graft vs. Host Disease,” “Toll-Like Receptor,” and “Neurotrophin” (Fig 7a), whereas IL1RN was linked to “Steroid Hormone Biosynthesis,” “Retinol Metabolism,” “Nod-Like Receptor Signaling Pathway,” and “Neurotrophin Signaling Pathway” (Fig 7b). In depression, DDIT3 was associated with “Ubiquitin-Mediated Proteolysis,” “Cell Cycle,” “Oxidative Phosphorylation,” and “Ribosome Pathway” (Fig 7c). IL1RN was associated with “Ribosome,” “Spliceosome,” “Fc Gamma R-Mediated Phagocytosis,” and “Acute Myeloid Leukemia pathways” (Fig 7d).

### Association between candidate biomarkers and immune infiltration

**Fig 8:**
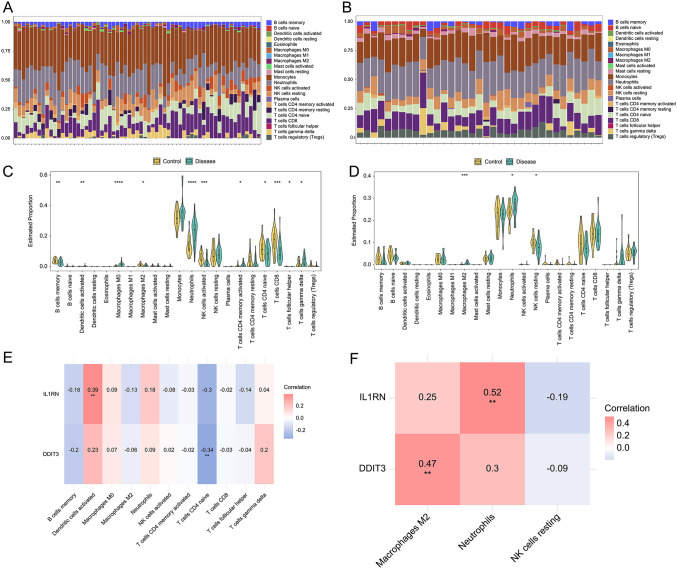
Comprehensive landscape of immune infiltration and its correlation with key biomarkers in ischemic stroke and depression. **(a)** Immune infiltration in the ischemic stroke training set assessed by CIBERSORT. X- and y-axes represent different samples and infiltration proportion, respectively. **(b)** Immune infiltration in the depression training set assessed by CIBERSORT. X- and y-axes represent different samples and infiltration proportion, respectively. **(c)** Differences in immune infiltration abundance between ischemic stroke disease and normal control samples. p-values estimated by the t-test: *p < 0.05, **p < 0.01, ****p < 0.0001. **(d)** Differences in immune infiltration abundance between depression disease and normal control samples. p-values estimated by the t-test: *p < 0.05, **p < 0.01, ****p < 0.0001. **(e)** Correlation between biomarkers and differentially infiltrated immune cells in ischemic stroke. X- and y-axes list immune cells and genes, respectively. Correlation coefficients and p-values estimated by the Spearman correlation test: *p < 0.05, **p < 0.01, ***p < 0.001. **(f)** Correlation between biomarkers and differentially infiltrated immune cells in depression. X- and y-axes list immune cells and genes, respectively. Correlation coefficients and p-values estimated by the Spearman correlation test: *p < 0.05, **p < 0.01, ***p < 0.001.

To assess immune infiltration in ischemic stroke and depression, the CIBERSORT algorithm evaluated the proportions of 22 immune cell types in disease and normal control samples from the training sets (Fig 8a, 8b). In ischemic stroke, infiltration of macrophages M0, neutrophils, T cells CD4^+^ memory activated, and T cells gamma delta was significantly increased in disease samples (Fig 8c). In depression, macrophages M2 and neutrophils exhibited significantly increased infiltration in disease samples (Fig 8d). Correlation analyses revealed that, in ischemic stroke, IL1RN exhibited a significant positive correlation with activated dendritic cells (p < 0.05), and both IL1RN and DDIT3 exhibited significant negative correlations with naive CD4^+^ T cells (p < 0.05) (Fig 8e, 8f). In depression, IL1RN was significantly positively correlated with neutrophils (p < 0.05), and DDIT3 was significantly positively correlated with macrophages M2 (p < 0.05).

### Signaling network open resource (SIGNOR) analysis of candidate biomarkers

**Fig 9:**
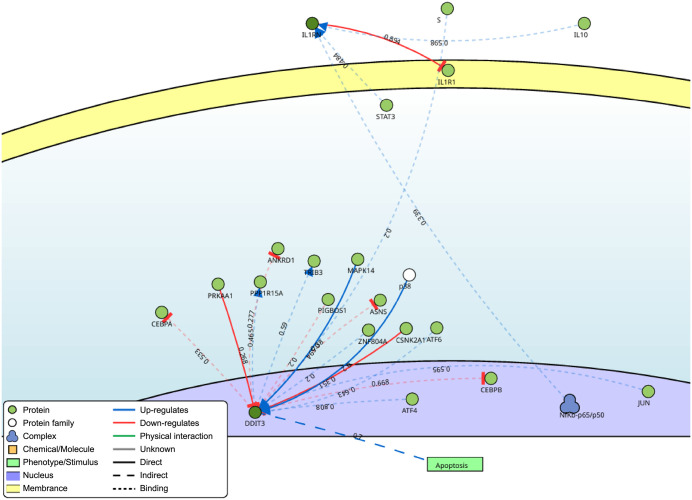
SIGNOR network of biomarkers with confidence scores ≥ 0.7. Red and blue indicate inhibitory and activating pathways, respectively.

The SIGNOR network analysis depicted potential regulatory interactions involving DDIT3 and IL1RN (Fig 9). DDIT3 exhibited interactions with multiple proteins and chemical entities, whereas IL1RN interacted only with STAT3, IL1R1, IL10, and NFκB-p65/p50.

### Molecular docking

**Fig 10:**
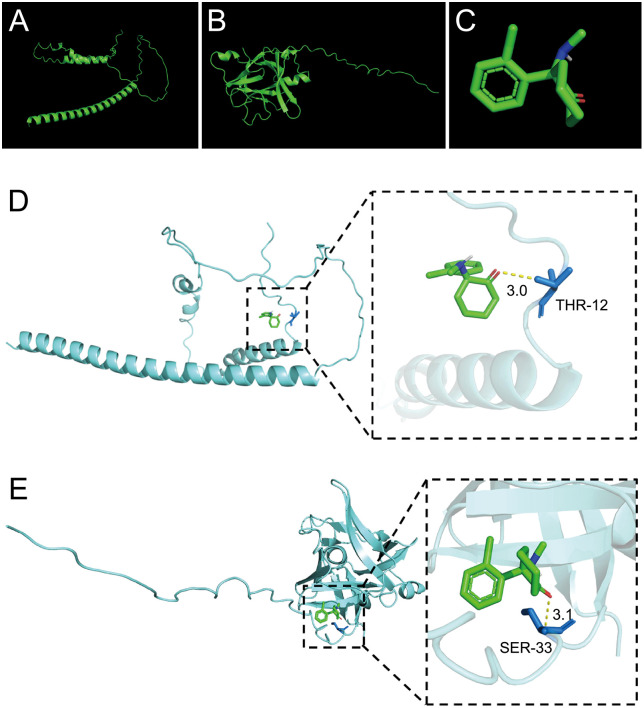
3D chemical structures of (a) DDIT3 and (b) IL1RN biomarkers. (c) 3D chemical structure of ketamine. (d) Molecular docking results for DDIT3 and ketamine. (e) Molecular docking results for IL1RN and ketamine.

Molecular docking was performed between candidate biomarkers and ketamine. The molecular structures of biomarkers and ketamine are shown in Fig 10a–10c, respectively. Docking results are shown in Fig 10d and 10e and Table 2. The binding energies were −4.5 and −5.8 kcal/mol for DDIT3 and IL1RN with ketamine, respectively, suggesting potential binding affinity.

**Table 2:** Molecular docking results.

Compound	Pubchem ID	Gene	Alphafold ID	(kcal/mol)
Ketamine	3821	IL1RN	P18510	−5.8
Ketamine	3821	DDIT3	P35638	−4.5

## Discussion

Depressive symptoms following stroke represent a common neuropsychiatric complication, often manifesting as anxiety, low mood, insomnia, and even suicidal ideation, affecting approximately one-third of stroke survivors [42]. This comorbidity significantly increases disability, recurrence, and mortality rates, emerging as an independent risk factor for poor prognosis. Nonetheless, safe and effective therapeutic agents specifically tailored for this condition are lacking [43,44]. In our clinical practice, we have observed the significant challenge posed by post-stroke depressive symptoms, which underscores the urgent need to investigate the underlying mechanisms of the ischemic stroke–depression comorbidity and explore novel treatments. Ketamine has garnered attention in this context owing to its unique pharmacological profile.

Recently, ketamine has been widely studied for treating TRD and patients with suicidal ideation owing to its rapid and sustained antidepressant effects, demonstrating marked superiority over conventional antidepressants in alleviating core depressive symptoms [23]. A single administration promotes the release of BDNF, activates the mTORC1 signaling pathway, increases synaptic protein levels, and enhances the number and function of synapses in the PFC, thereby rapidly reversing deficits in neuroplasticity [45]. Ketamine also exerts neuroprotective effects [46] by mitigating excitatory amino acid neurotoxicity via NMDAR blockade and by modulating the production and release of inflammatory factors, thereby reducing neuroinflammation. Furthermore, ketamine may improve post-injury cerebral hemodynamics by modulating vascular tone, enhancing regional cerebral blood flow, optimizing cerebral oxygen metabolism, and reducing ischemic–hypoxic brain damage [47]. However, despite these promising broad mechanisms, a systematic understanding of ketamine’s molecular targets and pathways in the specific context of comorbid ischemic stroke and depression remains largely elusive. Therefore, we employed a network pharmacology approach to explore and hypothesize potential targets of ketamine relevant to both conditions, aiming to generate novel strategic insights for future therapeutic development.

Intersecting predicted ketamine target genes with DEGs from ischemic stroke and major depressive disorder datasets yielded 42 intersection genes. KEGG and GO enrichment analyses suggested that these genes are potentially involved in pathways such as “Lipid and Atherosclerosis,” “IL-17 Signaling Pathway,” “Apoptosis,” “Response to Ketone,” “Response to Starvation,” and “Response to Oxygen Levels.” This implies that ketamine’s putative therapeutic effects might involve modulating lipid metabolism, inhibiting apoptosis, enhancing neuronal tolerance to ischemia and hypoxia, and maintaining immune homeostasis via suppression of the IL-17 signaling pathway. Subsequently, to interpret and rank the relative importance of these hub genes, SHAP-based interpretability analysis was performed. This approach does not aim to build diagnostic classifiers but to quantify each gene’s contribution to the model’s output, thereby identifying the most influential features. IL1RN and DDIT3 were prioritized as the top candidate biomarkers based on their consistently dysregulated expression patterns and robust discriminatory capacity in independent validation cohorts. To elucidate their potential functional roles, single-gene GSEA was performed. In the context of ischemic stroke, DDIT3 may be associated with immunity and inflammation via the Toll-like receptor signaling pathway, whereas both DDIT3 and IL1RN might be linked to neuronal survival and synaptic plasticity through the neurotrophin signaling pathway. In the depression dataset, DDIT3 may influence apoptosis and inflammation through ubiquitin-mediated proteolysis, oxidative phosphorylation, and ribosome pathway, whereas IL1RN could be involved in immune regulation via Fc gamma R-mediated phagocytosis.

IL1RN is an immunomodulatory molecule [48] that acts as an endogenous IL-1 receptor antagonist (IL-1Ra) [49], competitively binds IL-1R to inhibit signaling by IL-1α and IL-1β, thereby exerting anti-inflammatory effects. IL-1Ra suppresses proinflammatory cytokine production, potentially reducing tissue damage and maintaining immune balance [50]. Studies have reported increased IL1RN expression in post-stroke plasma [51], with plasma IL-1Ra serving as an independent predictor of post-stroke infection and conferring neuroprotection; administration of IL-1Ra has been shown to improve stroke outcomes in models [52]. Given that post-stroke depressive symptoms are frequently accompanied by neuroinflammation, our analysis leads us to hypothesize that ketamine might modulate IL1RN expression or its pathway, thereby suppressing IL-1β-mediated inflammation, mitigating neuroinflammation, and contributing to neural protection.

DDIT3 (CHOP) is a key regulator of endoplasmic reticulum (ER) stress, activated by DNA damage, hypoxia, and nutrient deprivation, and it modulates cell fate decisions including apoptosis. During ER stress, DDIT3 can promote apoptosis via the PERK–eIF2α–ATF4–DDIT3 pathway [53], activating BH3-only protein B-cell lymphoma 2 (BCL-2) interacting mediator of cell death (BCL2L11/BIM) and suppressing activity of anti-apoptotic protein BCL-2 [54]. Located in the nucleus and mitochondria, nuclear DDIT3 enhances glycolysis by inhibiting the negative regulator TIGAR, whereas mitochondrial DDIT3 downregulates COQ9 and COX4 to attenuate mitochondrial aerobic respiration. Thus, under hypoxic and nutrient-deficient conditions, DDIT3 maintains the energy balance by promoting glycolysis and suppressing mitochondrial oxidative phosphorylation, preventing the accumulation of harmful reactive oxygen species [55]. Additionally, ER stress mediates mitochondrial autophagy via the ATF4–DDIT3–TRIB3–AKT–mTOR pathway [56]. Therefore, we speculate that ketamine might influence DDIT3 expression or activity, potentially inhibiting apoptosis, maintaining metabolic balance, and modulating mitochondrial autophagy, collectively contributing to nervous system protection in the comorbid condition.

Immune dysregulation is implicated in both ischemic stroke and depression [57]. Our exploratory immune infiltration analysis revealed differences in immune cell proportions between disease and control samples. In ischemic stroke samples, increased infiltration of M0 macrophages, neutrophils, memory activated CD4^+^ T cells, and gamma delta T cells was observed. In depression samples, increased proportions of M2 macrophages and neutrophils were noted. Correlations were found between IL1RN and DDIT3 expression and specific immune cell types. After ischemic stroke, activated M0 macrophages effectively clear necrotic neurons from infarcted regions, reducing neuroinflammation and aiding neural recovery [58]. Post-stroke neutrophils exacerbate neuroinflammation through multiple mechanisms, including disruption of the blood–brain barrier, activation of the complement system, and release of inflammatory mediators, further inducing neuronal apoptosis or necrosis [59]. Memory activated CD4^+^ T cells migrate to brain injury sites, regulating immune responses via cytokine secretion [60]. Gamma delta T cells exhibit a dual nature in the nervous system by secreting cytokines that promote neuronal survival, growth, and differentiation, mitigate neuroinflammation, regulate the activity of other immune cells, and enhance blood–brain barrier stability to protect neural function. However, under certain pathological conditions, their excessive activation may provoke intense inflammation and autoimmune responses, aggravating secondary damage [61]. Post-stroke, activated dendritic cells rapidly migrate to the injured brain, participating in local inflammatory responses [62]. Moreover, type 2 dendritic cells induce IL-17 production by gamma delta T cells, further promoting neutrophil infiltration [60]. Meanwhile, in depression, M2 macrophages exert anti-inflammatory and neuroprotective effects by secreting cytokines such as IL-10, facilitating neural repair [63]. Neutrophils may contribute to depression onset by enhancing neuroinflammation, with studies reporting increased numbers and increased neutrophil activity in the peripheral blood and cerebrospinal fluid of patients with depression, correlating with chronic inflammation [64].

Molecular docking simulations suggested potential binding interactions between ketamine and the proteins IL1RN and DDIT3, with varying affinities. IL1RN exhibited the highest affinity, suggesting it may play a critical role in the efficacy of ketamine in PSD treatment. Collectively, our integrated network pharmacology analysis provides a hypothetical framework suggesting that the putative effects of ketamine in the context of ischemic stroke–depression comorbidity may involve modulating immune responses, reducing oxidative stress, inhibiting neuronal apoptosis, and regulating cellular metabolism.

Despite these insights, this study has some limitations that must be acknowledged. First, the analysis relies on public databases and computational predictions, which may not fully capture the complexity and dynamics of the disease state or drug action. The integrated set of ‘ketamine targets’ in this study was derived from public databases and encompasses associations ranging from direct binding targets to downstream regulatory genes. Although this broad strategy facilitates exploratory network analysis, the intersecting core genes should be interpreted as putative ketamine targets rather than definitive direct binding targets. Future experimental validation, including studies in relevant preclinical models and ultimately in clinical cohorts, is essential to confirm the roles of IL1RN, DDIT3, and the associated pathways. Second, the absence of in vitro or in vivo experimental data in this study confines it to the theoretical and hypothesis-generating level. Our hospital-based clinical setting provides a foundation for future translational research aimed at validating these candidate biomarkers in well-characterized patient cohorts. Finally, network pharmacology approaches are currently ill-equipped to assess critical pharmacological parameters such as dose–response relationships or long-term safety. Prolonged ketamine use may carry risks such as transient psychiatric symptoms, cognitive side effects, hemodynamic fluctuations, and dependency/abuse potential, necessitating careful clinical evaluation. The ultimate translation of these findings into clinical practice will require rigorous safety and efficacy trials in the target patient population.

## Conclusions

In conclusion, this study employed an integrated network pharmacology approach to explore the potential molecular mechanisms and candidate targets through which ketamine might exert effects in the context of comorbid ischemic stroke and depression. Our in silico analyses highlight IL1RN and DDIT3 as central candidate genes worthy of further investigation. We hypothesize that ketamine’s putative therapeutic actions may involve the modulation of multiple pathways—including IL-17 signaling, apoptosis, and cellular stress responses—potentially contributing to the suppression of neuroinflammation and apoptosis, enhancement of neuronal resilience, and maintenance of immune and metabolic homeostasis. These computational findings offer a novel, hypothesis-generating perspective on the pharmacological mechanisms of ketamine relevant to post-stroke neuropsychiatric sequelae. However, it is crucial to emphasize that these insights are derived from bioinformatic predictions and require rigorous validation. Future research should focus on experimental confirmation of these targets and pathways in relevant biological models, followed by clinical translation studies to assess their therapeutic relevance and safety in patient populations. This work provides a foundational framework for guiding such future investigations towards the development of more targeted therapeutic strategies.
